# Two decades of one health surveillance of Nipah virus in Thailand

**DOI:** 10.1186/s42522-021-00044-9

**Published:** 2021-07-05

**Authors:** Supaporn Wacharapluesadee, Siriporn Ghai, Prateep Duengkae, Pattarapol Manee-Orn, Weerapong Thanapongtharm, Abhinbhen W. Saraya, Sangchai Yingsakmongkon, Yutthana Joyjinda, Sanipa Suradhat, Weenassarin Ampoot, Bundit Nuansrichay, Thongchai Kaewpom, Rachod Tantilertcharoen, Apaporn Rodpan, Kachen Wongsathapornchai, Teerada Ponpinit, Rome Buathong, Saowalak Bunprakob, Sudarat Damrongwatanapokin, Chanida Ruchiseesarod, Sininat Petcharat, Wantanee Kalpravidh, Kevin J. Olival, Martha M. Stokes, Thiravat Hemachudha

**Affiliations:** 1 Thai Red Cross Emerging Infectious Diseases - Health Science Centre and WHO Collaborating Centre for Research and Training on Viral Zoonoses, King Chulalongkorn Memorial Hospital, Faculty of Medicine, Chulalongkorn University, Rama IV Road, Pathumwan, Bangkok, 10330 Thailand; 2 Forest Biology Department, Faculty of Forestry, Kasetsart University, Bangkok, Thailand; 3 Department of National Parks, Wildlife and Plant Conservation, Bangkok, Thailand; 4 Bureau of Disease Control and Veterinary Services, Department of Livestock Development, Bangkok, Thailand; 5 Department of Microbiology and Immunology, Faculty of Veterinary Medicine, Kasetsart University, Bangkok, Thailand; 6 Center of Excellence in Emerging and Re-emerging Infectious Diseases in Animals, Faculty of Veterinary Science, Chulalongkorn University (CU-EIDAs), Bangkok, Thailand; 7 National Institute of Animal Health, Department of Livestock Development, Bangkok, Thailand; 8 Program in Biotechnology, Faculty of Science, Chulalongkorn University, Bangkok, Thailand; 9 Food and Agriculture Organization of the United Nations, Bangkok, Thailand; 10 Department of Disease Control, Ministry of Public Health, Nonthaburi, Thailand; 11 U.S. Agency for International Development (USAID) Regional Development Mission for Asia, Bangkok, Thailand; 12 EcoHealth Alliance, New York, USA; 13 Defense Threat Reduction Agency, Biological Threat Reduction Program, Fort Belvoir, Virginia, USA

**Keywords:** Nipah virus, Outbreak, Thailand, One health, Surveillance, Pteropus

## Abstract

**Background:**

Nipah virus (NiV) infection causes encephalitis and has > 75% mortality rate, making it a WHO priority pathogen due to its pandemic potential. There have been NiV outbreak(s) in Malaysia, India, Bangladesh, and southern Philippines. NiV naturally circulates among fruit bats of the genus *Pteropus* and has been detected widely across Southeast and South Asia. Both Malaysian and Bangladeshi NiV strains have been found in fruit bats in Thailand. This study summarizes 20 years of pre-emptive One Health surveillance of NiV in Thailand, including triangulated surveillance of bats, and humans and pigs in the vicinity of roosts inhabited by NiV-infected bats.

**Methods:**

Samples were collected periodically and tested for NiV from bats, pigs and healthy human volunteers from Wat Luang village, Chonburi province, home to the biggest *P. lylei* roosts in Thailand, and other provinces since 2001. Archived cerebrospinal fluid specimens from encephalitis patients between 2001 and 2012 were also tested for NiV. NiV RNA was detected using nested reverse transcription polymerase chain reaction (RT-PCR). NiV antibodies were detected using enzyme-linked immunosorbent assay or multiplex microsphere immunoassay.

**Results:**

NiV RNA (mainly Bangladesh strain) was detected every year in fruit bats by RT-PCR from 2002 to 2020. The whole genome sequence of NiV directly sequenced from bat urine in 2017 shared 99.17% identity to NiV from a Bangladeshi patient in 2004. No NiV-specific IgG antibodies or RNA have been found in healthy volunteers, encephalitis patients, or pigs to date. During the sample collection trips, 100 community members were trained on how to live safely with bats.

**Conclusions:**

High identity shared between the NiV genome from Thai bats and the Bangladeshi patient highlights the outbreak potential of NiV in Thailand. Results from NiV cross-sectoral surveillance were conveyed to national authorities and villagers which led to preventive control measures, increased surveillance of pigs and humans in vicinity of known NiV-infected roosts, and increased vigilance and reduced risk behaviors at the community level. This proactive One Health approach to NiV surveillance is a success story; that increased collaboration between the human, animal, and wildlife sectors is imperative to staying ahead of a zoonotic disease outbreak.

**Supplementary Information:**

The online version contains supplementary material available at 10.1186/s42522-021-00044-9.

## Introduction

First identified during an outbreak in Malaysia in humans and swine in 1998, Nipah virus (NiV) is an emerging bat-borne zoonotic paramyxovirus that continues to re-emerge in humans in the South Asian region. NiV infection causes encephalitis and has > 75% mortality rate making it a World Health Organization (WHO) priority pathogen due to its pandemic potential [1–3]. Viral transmission of NiV to humans is either via contact or consuming blood or meat of an infected intermediary host (such as swine, horse), or directly through consumption of raw date-palm sap contaminated with bat excreta or consumption of fruits contaminated with saliva at the surface that were bitten by bats. Secondary transmission of NiV, i.e. human-to-human transmission, especially nosocomial transmission in different hospitals, or religious places have been reported in India and Bangladesh [4–8].

The natural reservoir of NiV is fruit bats of the family Pteropodidae. Thus, delving into their ecology and migration patterns, as well as surveillance studies at the bat-human interface near their roosts is central to understanding and staying ahead of a NiV outbreak [3, 9]. *Pteropus hypomelanus* and *P. vampyrus* were initially the putative natural reservoirs for NiV during the Malaysian outbreak in 1998. NiV has been detected in fruit bats, either by virus isolation or viral RNA from Malaysia, Bangladesh, India, Cambodia, Thailand, Indonesia, and East Timor [10–12]. NiV was then isolated from a *P. lylei* (or Lyle’s flying fox) bat roost in western Cambodia in 2000 [2, 13], and in Thailand in 2002, where viral RNA was detected by nested reverse transcription polymerase chain reaction (RT-PCR) and NiV IgG antibodies were detected in blood (9.3%) [2]. Additionally, serological evidence of NiV (or cross-reacting Nipah-like viruses) has been reported in bats from other families such as *Scotophilus kuhlii, Hipposideros Pomona,* and *H. larvatus* [2, 14, 15].

Diverse lineages of NiV have been detected across Asia [9]. Phylogenetic groups, or strains, of NiV has been labeled based on the country of the first outbreak and subsequently characterized as either Malaysia strain (NiV-MY) or Bangladesh strain (NiV-BD). Infectivity and pathogenicity of the two strains have been reported to be different in humans and experimented animals [16–22]. Additionally, it has been reported that clinical signs and viremia were undetectable, with very low levels of neutralizing antibody titers, in a study where pigs were inoculated with NiV-BD, unlike the response seen with NiV-MY infection. This suggests asymptomatic NiV-BD infected pigs could spread the virus undetected to susceptible pen mates, highlighting the need for continued and enhanced NiV surveillance in pigs in endemic regions [23].

NiV infection in humans or pigs in Thailand has not been reported to date (Thailand’s Ministry of Public Health (MOPH), Department of Livestock Development (DLD)). Both NiV-BD and NiV-MY strains have been detected in bats in Thailand. NiV-BD has been found in *P. lylei* roosting in central region of Thailand [2, 24]. NiV-MY was detected in *P. hypomelanus* in Southern Thailand, bordering Malaysia, thus NiV in fruit bats in Thailand is not limited to the central region or specific *Pteropus* species [16]. Genetic relationship amongst the Lyle’s Flying Fox populations were also examined in Central Thailand, which showed high levels of gene flow between the *P. lylei* colonies, and a high level of haplotype diversity in *P. lylei* population [25]. This extensive connectivity of *P. lylei* populations may help explain the continued circulation of NiV in *P. lylei* within 7 provinces in central Thailand [24].

GPS tracking of *P. lylei* bats between temples and orchards in central Thailand found that the maximum linear distances between day roosts and foraging areas varied greatly between individuals (2.2–23.6 km) but were similar between seasons [26, 27]. Knowledge of longitudinal, or seasonal patterns of NiV shedding in *Pteropus* bats can facilitate better understanding of the dynamics of NiV transmission. It was noted that viral shedding peaked in May at all sites, which coincided with physical separation between the mother and offspring [24]. In the last two decades, roost sites and population of Lyle’s Flying Fox has increased dramatically, from 16 roosts and 37,837 bats in 2004 to 30 roosts and 75,016 individual bats in 2017 [28].

Active One Health surveillance of NiV has been conducted in bats, humans, and pigs in Thailand since 2001 in collaboration with several laboratories and universities, the Ministry of Public Health, Ministry of Agriculture and Cooperatives, and Ministry of Natural Resources and Environment. This study reports on both published and previously unpublished results from 20 years of One Health surveillance in areas at high-risk of NiV emergence in Chonburi province, Thailand (Table 1). Chonburi province is where NiV was first found in *P. lylei* bat in 2003 and is home to the biggest *P. lylei* roost in communal property [2, 28]. Specimens were collected using a triangulated surveillance approach, with bats, pigs and humans sampled at the same location during 2003–2020 and tested for NiV viral RNA and NiV antibodies. Additionally, we describe educational tools used to raise community awareness regarding bats and their roles as hosts for NiV and ways to mitigate against NiV exposure.

**Table 1:** Timeline of NiV-related activities as part of the triangulated surveillance in the past two decades in Thailand

Year	Activity & Findings	Reference
HOST: BATS		
2000–2018	*An assessment of the niche centroid hypothesis: P. lylei (Chiroptera)* • We were unable to identify relationships between patterns of abundance using coarse-scale environmental variables but were able to identify relationships at a finer scale.	[29]
2002–2004	*Surveillance of NiV in bats in 9 provinces of Thailand, 1304 bats (12 species) were tested for PCR and serology.* • IgG Antibody seropositive: 7.8% (9.3% for *P. lylei*) • PCR positive: - Pooled saliva: 2/142 - Pooled urine: 6/142	[2]
2005–2007	*Surveillance of NiV in pooled urine collected under roosts of P. lylei bats in 7 provinces in central plains of Thailand* • NiV PCR positive from bat urine collected from all 7 provinces. • Seasonal prevalence transmission was found in April and May	[24]
2008–2012	*Annually collected bat pooled urine specimens from Chonburi* • NiV PCR positive was found in pooled urine ever year • % positive varied between 0 and 26% depending on number of samples collected, month. Note: there was possibility of duplicate positive from the same plastic sheet (urine from same bat could have been collected)	This study
2010–2011	*Surveillance of NiV in pooled urine of P. hypomelanus bats in southern Thailand* • NiV-MY strain was found in P. hypomelanus (PCR and sequencing).	[16]
2012	*Monthly capture of P. lylei bats at 2 pig farms and bat roost (*Fig. 1c*). NiV-PCR and serology conducted* • PCR positive bat saliva samples found in samples collected during Mar to Jul, highest number of positives in April. • NiV IgG antibody was found from bat throughout the year, except Jan (low number of tested bats)	This study
2012	*High-Resolution GPS Tracking of Lyle’s Flying Fox Between Temples and Orchards in Central Thailand* • Maximum linear distances between day roosts and foraging areas varied greatly between individuals (2.2–23.6 km) but were similar between seasons.	[26]
2014	*Genetic diversity and relationships among Lyle’s flying fox colonies in Thailand* • The sequence data suggested that the overall *P. lylei* population has high levels of haplotype diversity, which may reflect genetic exchange during *P. lylei* movement. These results will help manage populations and assess the risk of outbreaks of NiV carried by Lyle’s flying fox.	[25]
2015	*Spatial characterization of colonies of the flying fox bat, a carrier of NiV in Thailand* • Passive and active surveillance programs should be prioritized around Bangkok, particularly on farms with low biosecurity, close to water, and/or on which orchards are concomitantly grown. • Integration of human and animal health surveillance should be pursued in these same areas. • Such proactive planning would help conserve Lyle’s flying fox colonies and should help prevent zoonotic transmission of NiV and other pathogens.	[27]
2015–2016	*Assessing the distribution, roosting site characteristics, and population of P. lylei in Thailand* • The field survey validated the results of the questionnaire, with 67.65% of respondents correctly identifying *P. lylei* in their area • There were 30 roosting sites (8 new roosts), a total population of 75,016 bats, and a total roosting area of 1,328,720 m^2^ • Our results confirm that close proximity between *P. lylei* and human populations is common.	[28]
2015–2016	*Patch metrics of roosting site selection by Lyle’s flying fox (P. lylei) in a human-dominated landscape in Thailand* • The most suitable habitat areas for Lyle’s flying fox were associated with low patch contiguity, which was the most important spatial parameter affecting roosting site location	[30]
2016–2020	*Annually collected bat pooled urine specimens from Chonburi* • Whole genome of NiV was successfully sequenced from urine specimens collected from Chonburi in 2017. • NiV PCR positive was found in pooled urine ever year • % positive varied between 0 and 11.32% depending on number of samples collected, month. Note: there was possibility of duplicate positive from the same plastic sheet (urine from same bat could have been collected)	This study
HOST: PIGS		
2001 - present	*Annual NiV serosurveillance in pigs (4000–5000 pigs sampled each year)* • All pigs seronegative to date	National surveillance; DLD database
2011–2012	*A total of 246 pigs were sampled (246 nasal swabs, 233 serum samples) from 9 pig farms in Chonburi and Prachin Buri provinces where pig farms located close to (within 30 km radius) bat colonies where previously NiV-positive bats were found* • All nasal swabs tested negative for NiV RNA using PCR • All pig sera tested negative for NiV antibody using ELISA	This study
2016–2017	*A total of 1348 pigs were sampled from 36 pig farms on 3 sampling trips, in May 2016 (434 pigs), November 2016 (439 pigs), and February 2017 (475 pigs) in Chonburi and Chachoengsao provinces.* • All pig nasal swabs tested negative for NiV by PCR at the National Institute of Animal Health (NIAH) laboratory under the Department of Livestock and Development, Ministry of Agriculture and Cooperatives, Thailand. • NiV IgG antibodies were not tested in specimens from these sampling trips	This study
2019	*A spatial assessment of Nipah virus transmission in Thailand pig farms using multi-criteria decision analysis* • We believe that risk-based surveillance in the identified priority areas may increase the chances of finding NiV and other bat-borne pathogens and thereby optimize the allocation of financial resources for disease surveillance. • In the long run, improvements of biosecurity in those priority areas may also contribute to preventing the spread of potential emergence of NiV and other bat-borne pathogens.	[31]
2020	*A ‘what-if’ scenario: Nipah virus attacks pig trade chains in Thailand* • The risk of NiV dissemination through pig movement networks in Thailand is low but not negligible. • The risk areas identified in our study can help the veterinary authority to allocate financial and human resources to where preventive strategies, such as pig farm regionalization, are required and to contain outbreaks in a timely fashion once they occur.	[32]
HOST: HUMAN - Patient		
2001–2012	*Archived CSF specimens from encephalitis patients admitted at the King Chulalongkorn Memorial Hospital were tested for NiV PCR* • CSF from 232 encephalitis patients tested negative for NiV PCR	This study
HOST: HUMAN – Healthy Volunteers from high-risk community		
Nov-Dec 2010	*A total of 418 serum of local residents (voluntary participation; informed consent was sought) in Wat Luang village, Chonburi province, were collected where NiV-positive bats were previously found in the bat colonies* • Blood samples were assayed for IgG antibodies using indirect ELISA against NiV-infected cell lysate. • No NiV-specific IgG antibodies were found.	This study
May 2017 & May 2018	*Samples (oral swabs, urine, and serum) were tested for NiV and NiV antibodies in May 2017 and May 2018 (115 and 128 subjects were enrolled respectively)* • All oral swabs and urine specimens tested negative for NiV using PCR. • All serum specimens tested negative for NiV antibodies using Luminex serology assay.	This study

## Materials and methods

### Sampling

**Fig. 1:**
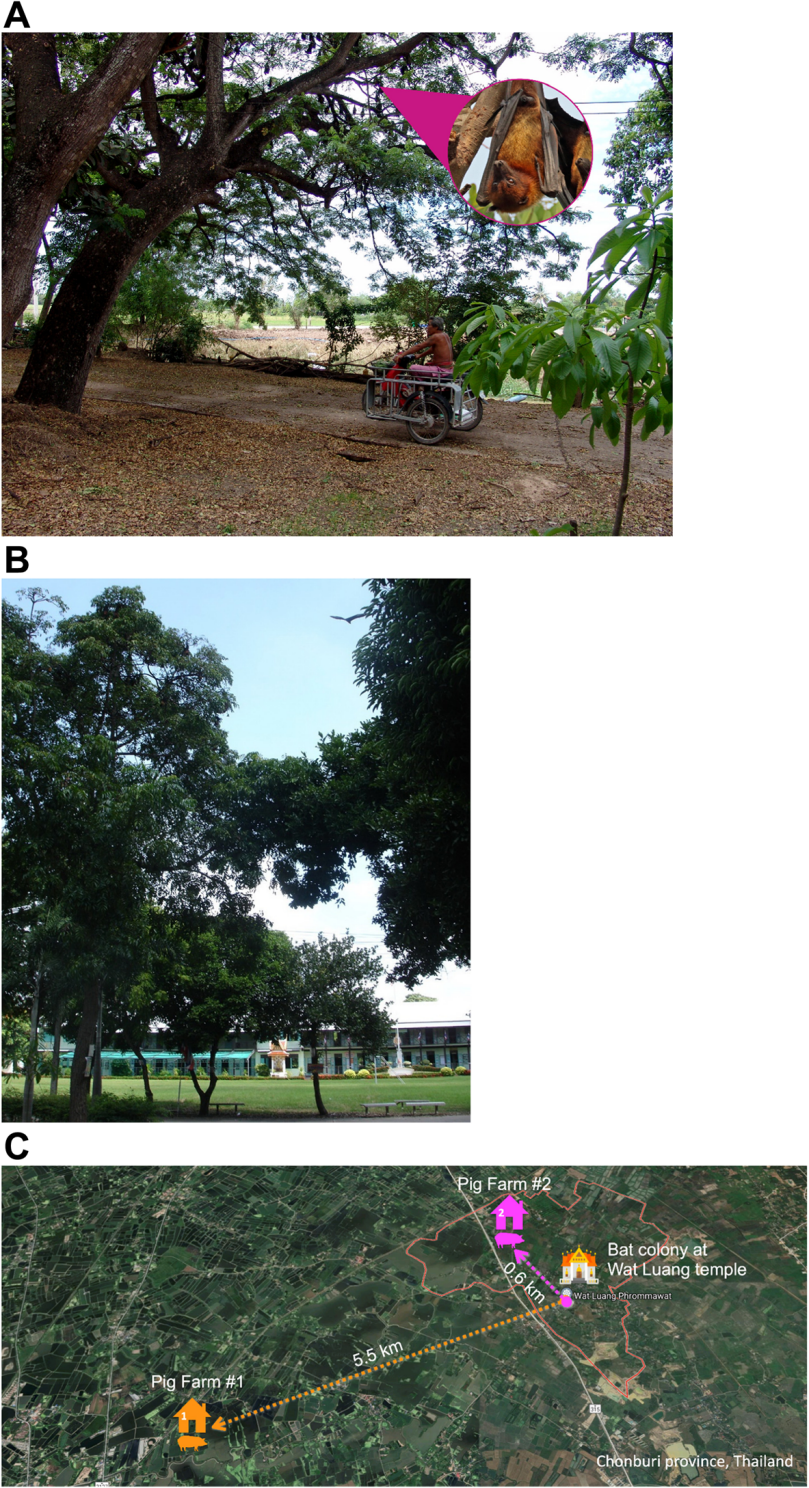
**a** Wat Luang village resident passing under a bat roosting tree in Chonburi province, Thailand. **b** One of the many bat roosting trees at Wat Luang Primary School, Wat Luang village, Chonburi Province, Thailand. A bat (often mistaken for a bird by people) can be seen flying away from the tree in broad daylight. **c** The three main study sites in Chonburi province, Thailand included in this study: Bat colony at Wat Luang temple, Wat Luang village, Pig Farm #1–5.5 km from Wat Luang temple, and Pig Farm #2–0.6 km from Wat Luang temple

Wat Luang village, Chonburi province was selected as the study site as it houses the biggest *P. lylei* colony in Thailand where > 10,000 and 7991 bats were found in 2006 and 2016, respectively [24, 28], and people in the community are considered high-risk for NiV infection due to the human-bat-pig interfaces (see Fig. 1). Sites within 30 km of Wat Luang were considered high-risk to account for the distance *P. lylei* travel to forage (at least 23 km) [26, 27]. This study was mainly a collaboration between the Faculty of Medicine & Faculty of Veterinary Science, Chulalongkorn University; Faculty of Forestry and Faculty of Veterinary Medicine, Kasetsart University; Department of Disease Control, MOPH; DLD under the Ministry of Agriculture and Cooperatives; and the Department of National Park, Wildlife and Plant Conservation (DNP) under the Ministry of Natural Resources and Environment.

#### Surveillance in bats

##### Pooled bat urine sampling

Pooled bat urine samples were collected almost every year since 2009 (except 2013–2015) using plastic sheets placed under bat roosting trees as previously reported [19, 24]. Thirty plastic sheets were placed under the trees; 2–4 sheets/tree depending on the number of bats at each spot. Each sheet was 1.5 × 1.5 m in dimension. One mL of urine was suctioned using cotton swab or sterile pasture pipette and added into 9 mL of NucliSENS Lysis buffer containing guanidine thiocyanate and Triton X-100 (bioMérieux, Marcy-l’Étoile, France). Total of at least 50 tubes of urine specimens were collected and placed in an insulated box packed with frozen refrigerant packs during each sampling trip and transported to the testing laboratory within 24 h.

##### Bat capture

*P. lylei* bats were captured two nights a month in 2012 at three sites: bat colony at Wat Luang temple, and 2 pig farms within 30 km of the temple (Fig. 1c). At least 10 bats from the bat colony were captured each visit, and bats at the pig farms were trapped using mist-nets on the same nights. Bats were not euthanized and were released after measurements were taken and samples were collected. Oral swab was collected from individual bats and immediately put into Lysis buffer (bioMérieux, Marcy-l’Étoile, France) for further testing for NiV RNA by nested RT-PCR. Blood was collected from bat wing vein for further serology testing for NiV antibody.

#### Surveillance in pigs

DLD conducts annual NiV serosurveillance in pigs (4000–5000 pigs sampled each year) from pig farms around the country since 2001. Additionally, in 2011–2012, pig sera and nasal swabs were collected from pig farms located within 23 km of Wat Luang temple by the team from Faculty of Veterinary Science, Chulalongkorn University. In 2016–2017, pig sera and nasal swabs were collected from farms within 30 km of Wat Luang temple by DLD. The samples were transported on ice to laboratory within 48 h. The samples were stored at -80 °C until further analysis. Pig nasal swabs were tested for NiV RNA by nested RT-PCR. Pig sera were tested for NiV antibody by enzyme-linked immunosorbent assay (ELISA).

#### Surveillance in humans

##### Patient samples: cerebrospinal fluid (CSF) specimens

Archived CSF specimens from 243 encephalitis patients admitted to the King Chulalongkorn Memorial Hospital between 2001 and 2012 were tested for NiV RNA using nested RT-PCR.

##### Human samples: serum, oral swab, and urine specimens

Blood, oral swabs, and urine samples were collected from healthy volunteers who worked, lived, or visited Wat Luang village in Chonburi province (Wat Luang) where NiV-infected bats were previously found [2, 24]. Only blood was collected from volunteers enrolled in 2010, while blood, oral swabs and urine samples were collected from volunteers in May 2017 and May 2018. Information sheet was provided, and consent forms were signed by the healthy volunteers before sample collection. Blood serum was used for antibody detection by ELISA or multiplex microsphere immunoassay (MMIA). Urine and oral swab samples were tested for NiV RNA using nested RT-PCR.

Thai Red Cross Emerging Infectious Diseases Health Science Centre (TRC-EID-HSC) further raised community awareness amongst the residents of Wat Luang village regarding bats and their roles as hosts of NiV and other bat-borne pathogens and provided ways to mitigate against NiV exposure. Educational booklet, “Living Safely with Bats”, a USAID-developed teaching aid tailored to the non-scientist population was translated and distributed by TRC-EID-HSC to Wat Luang village residents and Health Promoting Hospitals.

### Laboratory testing

#### Nested RT-PCR to detect NiV RNA

Samples from bats, humans and pigs from this study were tested for NiV using the duplex-nested RT-PCR method. Briefly, total nucleic acid extraction was performed using the easyMAG kit (bioMérieux). NiV RNA was detected by nested RT-PCR using specific NiV primers previously described [33]. PCR positive specimens were further characterized for NiV sequence to identify viral strain using heminested RT-PCR. Heminested amplification was performed from the first-round PCR product and the nucleotide sequences of N-gene at positions 1197–1553 (according to GenBank Accession No. NC_002728) were analyzed as described previously [33].

#### Next generation sequencing (NGS) for whole genome sequencing (WGS)

##### Library preparation

The extracted nucleic acid previously positive by nested RT-PCR was treated with DNase I and used for DNA library preparation using TruSeq stranded total RNA library preparation kit. The prepared library was run with the MiSeq reagent kit V.3 (Illumina, USA) on the Illumina MiSeq machine. Two methods were used for WGS assembly:

##### Reference mapping analysis pipeline

The obtained nucleotide sequences were analyzed and mapped to the reference sequence followed by top-hit Blast result downloaded from GenBank. The WGS was assembled using Burrows-Wheeler Aligner (BWA) software [34], the consensus sequence was generated using SAMtools [35] and the WGS was visualized by Tablet, an NGS assembly visualizer [36].

##### Non-reference genome metagenomic analysis pipeline (NGMAP) from IDBA-UD toolkit for de novo assembly

The obtained nucleotide sequences were analyzed using NGMAP by de novo assembly and mapped to the reference sequence followed by top-hit de novo assembly result. The taxonomy for the contigs from the de novo assembly step were identified by BLAST tool. The WGS was assembled by Bowtie2 [37], consensus sequence generated using SAMtools [35], and the WGS was visualized by Tablet visualizer [36].

#### Phylogenetic analysis

NiV sequence data from surveillance and molecular detection between from 2002 to 2020 were combined for phylogenetic analyses. The WGS or partial genome was aligned using MAFFT [38]. Phylogenetic tree was constructed using the maximum-likelihood method, general time-reversible (GTR) substitution model and bootstrap test with 1000 replicates by MEGA X [39]. Phylogenetic tree was visualized with FigTree V 1.4.2, using single bootstrap test with 1000 replicates [40].

#### Antibody assay

##### Enzyme-linked immunosorbent assay (ELISA): human and pig samples (2012)

Pig and human sera collected in 2012 were screened for NiV antibodies by ELISA at the National Institute of Animal Health (NIAH), DLD, and the TRC-EID-HSC respectively. The antigens, inactivated NiV-infected Vero E6 cells, was kindly provided to TRC-EID-HSC by the Viral Special Pathogens Branch, US Centers for Disease Control and Prevention. Sera were diluted 1:100 with diluent and four-fold dilutions were made from 1:100, 1:400, 1:1600, and 1:6400. Peroxidase-labeled protein A/G or anti-human IgG (Pierce, Rockford, IL, USA) was used as conjugate for animal and human antibody testing, respectively. The cutoff OD value was 0.2. Three negative control serum samples and positive control samples were included in each run. Positive criteria for NiV infection was a dilution of ≥1:400.

#### Pan-henipavirus MMIA: human samples (2018)

The presence of immunoglobulins against henipavirus, a soluble native-like oligomeric virus envelope glycoproteins (which was kindly provided to the TRC-EID-HSC by the Department of Microbiology and Immunology, Uniformed Services University, USA), was measured using pan-henipavirus MMIA. Briefly, soluble tetrameric henipavirus receptor binding proteins (sG_tet_) were produced, as described elsewhere [41]. Purified sG_tet_ and envelope glycoprotein antigens were coupled to Bio-Plex Pro magnetic COOH beads (Bio-Rad, California, USA). Serum samples were heat inactivated at 56 °C for 30 min and diluted 1:250 before screening, and each sample was run in duplicates.

## Results

### Surveillance in bats

#### Longitudinal study of NiV from pooled bat urine samples

**Fig. 2:**
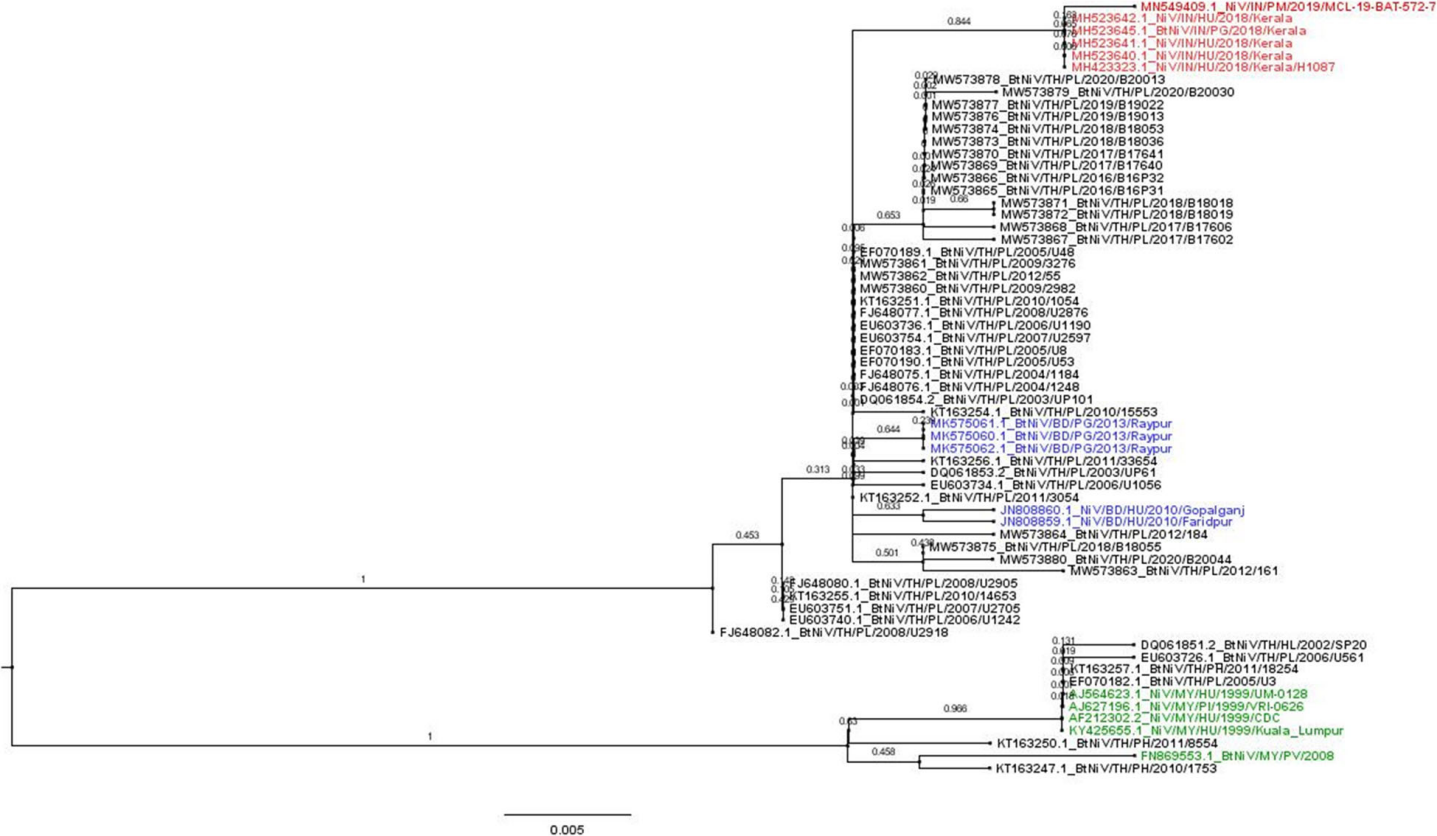
Phylogenetic tree of Nipah virus (NiV) genes found in *P. lylei* bats in Thailand: partial coding sequence (cds) of nucleocapsid (N) gene (357 bp). NiV positive sequences from this study and previous reports, from 2002 to 2020, were analyzed. Maximum-likelihood method was used to analyze NiV phylogeny based on the Kimura 2-parameter model. Bootstrap values were determined using 1000 replicates via MEGA X. Forty-seven NiV found in Thailand (21 from this study) were selected for phylogenetic analysis. Identical NiV from *P. lylei* were not included in the analysis. Thai NiV is shown in black (Full list can be found in Supplementary Table 1), while the reference sequences used in this study are shown in red (India), blue (Bangladesh) and green (Malaysia) respectively. BD = Bangladesh, IN = India, TH = Thailand, MY = Malaysia; PG = *Pteropus giganteus*, PL = *Pteropus lylei*, PH = *Pteropus hypomelanus*, HL = *Hipposideros larvatus*, HU = Human, PI = Pig, Bt = Bat

A total of 139 from 2500 pooled urine specimens collected from *P. lylei* bats from 2002 to 2020 were PCR-positive for NiV (Table 2). NiV-positive *P. lylei* bats were found every year, indicating that the virus circulating in this bat species served as the natural reservoir of NiV in Thailand. The NiV RNA positive samples from 2003 to 2020 were further sequenced for phylogenetic study of NiV found in Thailand. The Thai NiV isolates formed a monophyletic group with both NiV-MY and NiV-BD strains (Fig. 2). The majority of NiVs collected from *P. lylei* from Wat Luang village, Chonburi province, were NiV-BD. NiV sequences from 2003 to 2020 shared 99.44 to 100.00% nucleotides identity among themselves (355–357/357 nucleotide base pairs (bp)).

**Table 2:** Nested RT-PCR results detecting NiV RNA from pooled urine specimens from *Pteropus* bats collected during 2002 to 2020 in Thailand

Location	Bat Species^a^	Collection Date	No. of tubes tested	No. of tubes positive	NiV RNA prevalence Positive/Tested (%)	Ref^c^
Kram Chon	PH	2002	7	0	0.00	[2]
Surat	PV	2003 Oct	5	0	0.00	[2]
4 sites^b^	PL	2003 Jan - Sep	25	2	8.00	[2]
Singburi, Ayutthaya	PL	2002	31	0	0.00	[2]
Chonburi	PL	2003 Feb	16	0	0.00	[2]
		2004 Feb	15	4	26.67	[2]
		2005 Apr	50	1	2.00	[24]
		2005 May	50	2	4.00	[33]
		2005 May	48	6	12.50	[24]
		2005 Jun	31	1	3.23	[24]
		2006 Jan	37	1	2.70	[24]
		2006 Apr	80	3	3.75	[24]
		2006 May	51	4	7.84	[24]
		2006 Jun	59	1	1.69	[24]
		2007 Feb	49	3	6.12	[24]
		2007 May	99	4	4.04	[24]
		2005–2007	683	0	0.00	[24]
	Bat	2008 Apr	100	6	6.00	[42]
		2009 Apr	100	9	9.00	This study
		2010 Feb	47	0	0.00	
		2010 Apr	113	24	21.24	
		2011 Apr	129	28	21.71	
		2012 Mar	50	7	14.00	
		2012 Apr	50	13	26.00	
		2016 May	50	2	4.00	
		2016 Nov	50	0	0.00	
		2017 Feb	75	0	0.00	
		2017 May	100	4	4.00	
		2017 Nov	47	0	0.00	
		2018 Feb	75	0	0.00	
		2018 May	75	5	6.67	
		2019 Apr	53	6	11.32	
		2020 May	50	3	0.00	
Total			**2500**	**139**	**5.56%**	

^a^*PH Pteropus hypomelanus*, *PV P. vampyrus*, *PL P. lylei*; ^b^4 sites: Chachoengsao, Rayong, Prachin Buri, and Bangkok provinces; ^c^Previously published

#### WGS of NiV from a pooled bat urine sample

The NiV positive bats’ pooled urine sample (*P. lylei*) collected in May 2017 was further characterized for WGS using NGS technology. Sequence was analyzed using two protocols: reference mapping protocol with NiV from Bangladesh 2004 patient as a reference; NGMAP de novo assembly step from IDBA_UD from EDGE Tools. Total of 18,326 nucleotides of WGS were obtained from both protocols which were identical to each other. The phylogenetic tree was constructed, which showed the WGS from NiV isolates from Thailand formed monophyletic group with those from humans and bats *(P. medius*) in Bangladesh.

**Fig. 3:**
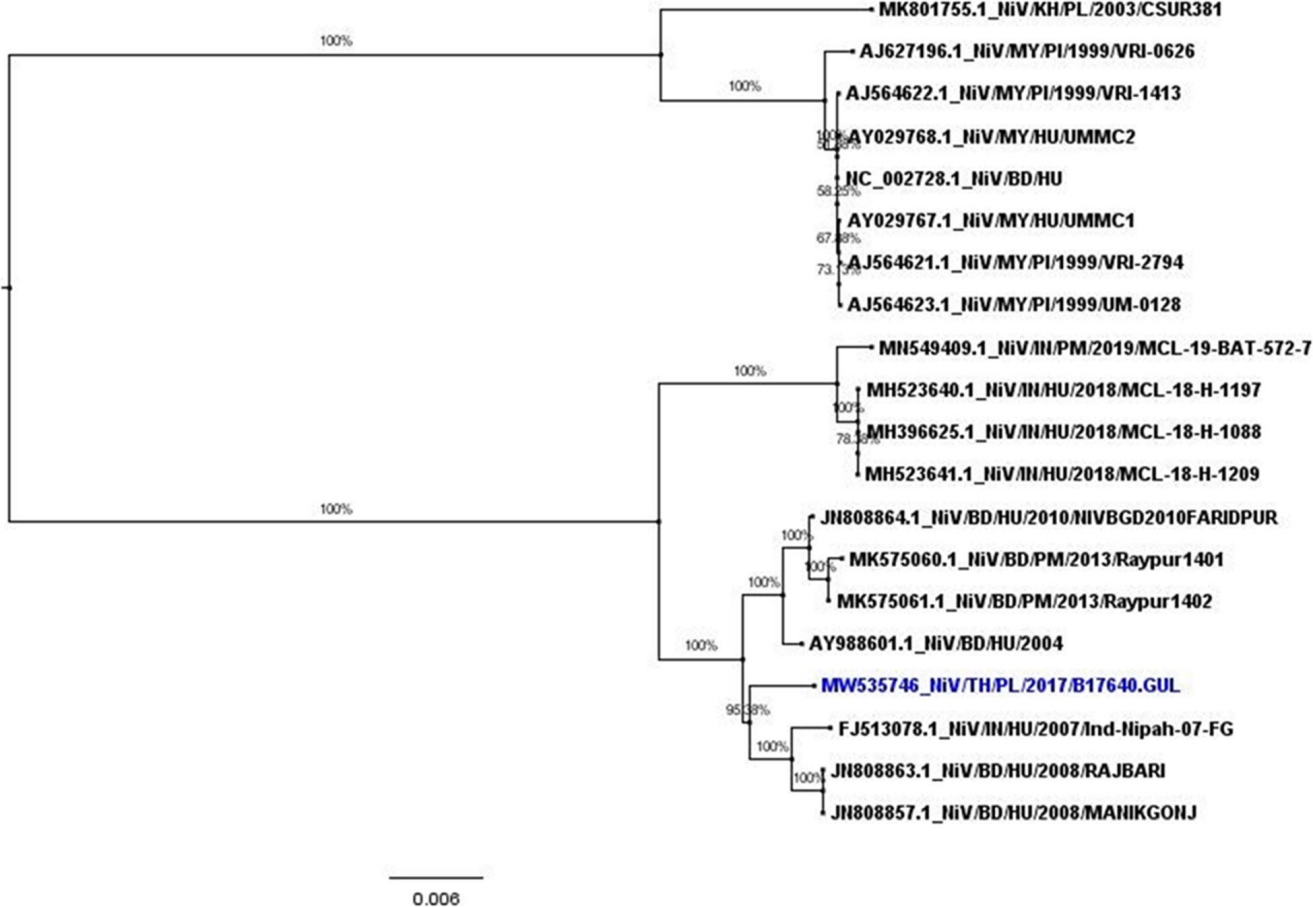
Phylogenetic tree analysis of NiV whole genome sequence (18,127 nucleotides each) isolated from *P. lylei* in Thailand in 2007 (shown in blue). Maximum-likelihood method was used to analyze phylogenetics, using sequences from pigs, bats, and humans available in GenBank (shown in black). HU = Human; PI = Pig; PL = *P. lylei*; PM = *P. medius;* MY = Malaysia; BD = Bangladesh; IN = India; KH = Cambodia; TH = Thailand

The genome data was deposited to NCBI GenBank (Accession No. MW535746). Phylogenetic tree of the NiV WGS (Fig. 3) showed that the virus was very closely related to NiV from Bangladeshi patient in 2004 (AY988601.1), Indian patient in 2007 (FJ513078.1) and bat in Bangladesh (*P. medius*) in 2013 (MK575061.1) (99.13%, 99.03 and 98.97 shared nucleotides, respectively) than the Malaysian patient in 1999 (AJ564622.1) (91.58% shared nucleotides). The amino acid identity among 6 genes of Nucleocapsid (N), Phosphoprotein (P), Matrix protein (M), Fusion protein (F), Glycoprotein (G) and RNA Polymerase (L) were 99.81, 99.44, 100.00, 98.90, 99.83% and, 99.55%, respectively (Table 3). Furthermore, the virus shared 97.82% (17,813/18210 nucleotides, 397 nt difference) with NiV isolated from an Indian patient from the recent outbreak in 2018 (GenBank Accession No. MH396625).

**Table 3:** Identity shared between THAB17640.GUL with NiV from Bangladesh patient in 2004 (GenBank Accession No. AY988601.1)

Target gene	Amino acid sequence range	Nucleotide sequence range	% Identity of Amino acid	% Identity of Nucleotide
Nucleocapsid (N)	1–532	56–2297	99.81 (531/532)	99.29 (2226/2242)
Phosphoprotein (P)	1–709	2301–5004	99.44 (705/709)	99.59 (2693/2704)
Matrix protein (M)	1–352	5108–6366	100.00 (352/352)	98.81 (1244/1259)
Fusion protein (F)	1–546	6370–8712	98.90 (540/546)	98.85 (2316/2343)
Glycoprotein (G)	1–602	8716–11,261	99.83 (601/602)	98.98 (2520/2546)
RNA Polymerase (L)	1–2244	11,265–18,219	99.55 (2234/2244)	99.12 (6894/6955)

#### Longitudinal study of NiV RNA and IgG antibody from *P. lylei*

**Fig. 4:**
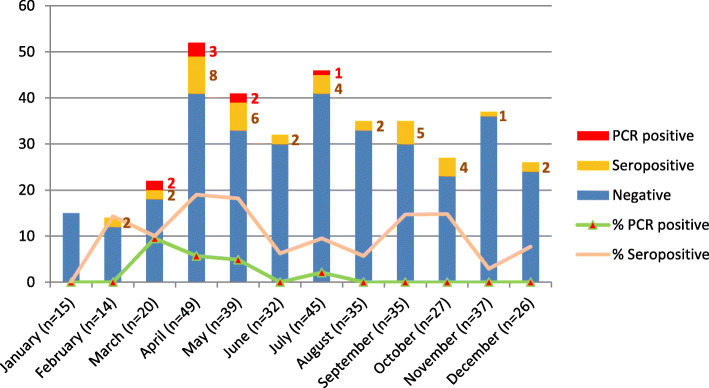
Nested RT-PCR and antibody results of specimens from *P. lylei* bats (*N* = 374) collected monthly in 2012. Number of bats captured each month (indicated), and number of PCR-positive and serology-positive bats are indicated in blue, green and yellow in the bar graph, respectively. Percent of PCR-positive and seropositive bats are indicated by green and pink line, respectively

A total of 374 bats (15–49 bat/month) were captured monthly from 3 study sites in Chonburi province in 2012. Eight of 374 (2.14%) of bat saliva samples (oral swabs) were positive for NiV RNA by nested RT-PCR. Two, 3, 2, 1 NiV positive *P. lylei samp*les were found in March, April, May, and July of 2012, respectively (Fig. 4). Three of 8 positive saliva specimens were collected from bats trapped at Pig Farm #2 (Fig. 1c). Positive specimens were further sequenced to identify the NiV strain. All eight were NiV-BD.

Bat sera (*n* = 374) were tested for IgG antibody using ELISA methodology provided by the US-CDC. NiV antibody positive sera were found in bats from all 3 study sites; 2/105 (1.90%), 5/51 (9.80%) and 31/218 (14.22%) from Pig Farms #1, #2, and bat colony at Wat Luang temple, respectively (Fig. 1c). Thirty-eight of 374 (10.16%) were positive for NiV antibodies throughout the year, except January (Fig. 4).

### Surveillance in pigs

#### Study conducted during August 2011 to November 2012

A total of 246 pigs were sampled from 9 pig farms in Chonburi and Prachin Buri provinces where pig farms located close to (within 30 km radius) bat colonies where previously NiV-positive bats were found between August 2011 and November 2012. All pig nasal swabs were negative for NiV-RNA by nested RT-PCR, while all 233 pig sera collected tested negative for NiV antibody by ELISA (Supplementary Table 2).

### Study conducted during May 2016 to February 2017

A targeted risk-based surveillance in pigs was conducted during May 2016 to February 2017. A total of 1348 pigs were sampled from 36 pig farms located closely to NiV-positive bat colonies on 3 sampling trips in May 2016 (434 pigs), November 2016 (439 pigs), and February 2017 (475 pigs) in Chonburi and Chachoengsao provinces. All pig nasal swabs tested negative for NiV by nested RT-PCR. The test was performed by NIAH under DLD. Serological test for NiV IgG antibodies were not included in this study. However, Thailand shares border with Malaysia and the sporadic emergence of human cases in Bangladesh and India, DLD established annual NiV serosurveillance in pigs since 2001. To date, all pigs are seronegative to NiV IgG antibodies indicating that NiV does not circulate in pig populations around the country.

### Surveillance in human

During the sample collection trips, results from previous NiV cross-sectoral surveillance were conveyed to villagers which led to preventive control measures, increased vigilance and reduced risk behaviors at the community level. One hundred community members were trained on how to live safely with bats.

#### Study conducted in archived CSF samples from encephalitis patients

Two hundred and forty-three CSF specimens from encephalitis patients admitted to the King Chulalongkorn Memorial Hospital between 2001 and 2012 tested negative for NiV RNA using nested RT-PCR.

#### Study conducted during November to December 2010

A total of 418 serum samples of local residents (voluntary participation, and informed consent was sought) in Wat Luang village, Chonburi province, were collected between November and December 2010, where NiV were previously found in the bat colonies (high-risk communities). Serum samples were assayed for IgG antibodies using indirect ELISA against NiV-infected cell lysate. All serum samples from the high-risk community were seronegative to NiV-specific IgG antibodies.

#### Study conducted during May 2017 and May 2018

Samples (oral swabs, urine, and serum) from healthy humans living in high-risk communities were collected twice, in May 2017 and May 2018 (115 and 128 subjects enrolled, respectively) were tested for NiV and NiV antibodies (Supplementary Table 3). All oral swabs and urine specimens were negative for NiV using nested RT-PCR. All serum specimens were seronegative for NiV antibodies using Luminex serology assay.

## Discussion

A confluence of risk factors for NiV disease outbreaks occur in Thailand, notably the regular detection of NiV in *Pteropus* bats, presence of three NiV primary natural reservoirs species (*P. lylei, P. hypomelanus, and P. vampyrus*) which are found in communities and tourist areas in central and southern Thailand [2, 16], and pig farming in areas within flight range of *Pteropus* roosts. This study shows that *P. lylei* urine has been positive for NiV at the same location for 18 years, which indicates *P. lylei* is a natural reservoir for NiV in Thailand and the virus is shed annually. *P. lylei* inhabits the human-dominated areas in Thailand (53% of roost sites are located within Buddhist temples, while the number of roosts located on private, common, and state properties are 8 (27%), 3 (10%), and 3 (10%), respectively. There were few ecological limits of movement of *P. lylei* in the central plains of Thailand [28]. Further, according to the DLD, a total of 12,228,255 pigs were recorded nationwide in 2020, an overall high density for a known intermediary host of NiV, [43]. However, most breeding pig farms are raised in closed systems, especially in the Central region of Thailand, thus effectively reducing the risk of NiV transmission from bats to pigs. Additionally, a recent study predicted additional “likely reservoirs” of NiV using machine learning based on traits using several parameters [14]. Thus, increased vigilance and collaboration between the human, animal, and wildlife sectors is imperative to staying ahead of a zoonotic disease outbreak.

In this study, we compiled findings from 20 years of proactive, multi-sectoral One Health surveillance, and research to facilitate preparedness for NiV prevention and control in Thailand. This included NiV serosurveillance in pigs since 2001, surveillance in bats since 2002, encephalitis patients from 2001 to 2012, and among healthy high-risk community volunteers in 2010, 2017 and 2018 (Table 1).

In the long-term surveillance of NiV in *P. lylei* bats in Thailand, NiV RNA has been detected in pooled urine samples every year since 2003 to 2020 (data from this study and published data [2, 24, 33, 42]) (139/2500, 5.56%; Table 2). This is a significantly higher rate than previous detections in Cambodia from 2012 to 2016 in the same bat species, which found 28 NiV-positive urine samples (from 3930; 0.7%) by PCR [11]. Both NiV strains were found from *P. lylei* in Thailand however, NiV-BD was dominant (Fig. 2). The WGS of NiV RNA, sequenced directly from pooled bat urine specimens from Chonburi in 2017, shared 99.13% nucleotide identity to NiV from a Bangladeshi patient in 2004 (Genbank Accession no. AY988601.1). On the contrary, previous WGS of NiV from Cambodian (MK801755.1) bats were NiV-MY strain. Overall homology of naturally circulating NiV strains in Thailand with previously detected human infectious strains from Bangladesh highlights the outbreak potential of NiV from *P. lylei* to humans or pigs in the Southeast Asian region.

Routine NiV serosurveillance in pigs in Thailand has been conducted annually since 2001 by DLD. At least 4000–5000 pig sera are collected annually around the country for NiV antibody testing by ELISA at high-risk sites selected by DLD on the basis of bat NiV studies [2]. To date, no NiV-seropositive pig has been identified from this DLD initiative [27]. In this study, two targeted risk based NiV surveillances in pigs were conducted. The first study, from August 2011 to November 2012, was conducted by the researchers from the Faculty of Veterinary Science, Chulalongkorn University, where 246 and 233 specimens were negative for antibody testing in serum and NiV RNA testing in nasal swabs, respectively. The second study was conducted by DLD researchers from May 2017 to May 2018; all 1349 pig specimens tested negative for NiV RNA by PCR. The results of these two studies were similar to the studies conducted in Laos and Indonesia. In Laos during May 2008 to January 2009, 716 pig sera tested negative for NiV [44]. In the Indonesian study, all 610 pig sera tested negative despite 19% of seropositive *P. vampyrus* to NiV antibodies [45]. Additionally, the study by Kasloff et al. highlighted that pigs infected with NiV-BD did not show clinical signs, nor viremia, which emphasizes the need for laboratories and pig farm owners to remain vigilant [23]. Together these findings suggest that bat to pig spillover of NiV is a rare event, especially given surveillance of healthy pigs.

In Malaysia, NiV serosurveillance among 177 indigenous, healthy volunteers from 4 communities, located 30–75 km from either previously confirmed NiV infections or locations where *Pteropus* bats were seropositive to NiV antibodies [46]. It was found that 10.73% of the participants had antibodies against NiV nucleocapsid protein, suggesting possible exposure to NiV. To date there is no evidence of NiV infection in humans or pigs in Thailand. To identify potentially undetected spillovers of NiV from *P. lylei* to humans in the past, serosurveillance in community was conducted at the village where *P. lylei* bats were positive to NiV RNA and NiV antibodies [2, 24]. The first serosurveillance was conducted in November and December 2010, where 418 sera from people in the village tested negative by ELISA (using inactivated virus as the antigen). Oral swabs and urine samples from the villagers collected in May 2017 (*n* = 115) and May 2018 (*n* = 128) tested negative for NiV RNA. Serum samples of healthy villagers collected in 2018 (n = 128) further tested negative for NiV antibodies using Luminex assay. The timing of our human community-based surveillance (the month of May) corresponded to the highest prevalence of NiV RNA detected in bat pooled urine at this site (Wat Luang) [24]. No evidence of NiV infection was found in the villagers at the study sites during this viral shedding period. Our findings on community serosurveillance are similar to a study in Cambodia where NiV has been reported in *P. lylei*, yet none of the 418 potentially exposed people were seropositive for NiV [11].

The low prevalence of NiV (less than 10% NiV RNA) and the seasonal prevalence pattern in *P. lylei* found in Thailand and Cambodia might reduce the risk of NiV transmission from bats to humans, in addition to the absence of key risk behaviors associated with viral transmission. From our assessment, drinking of raw date palm juice is not common in Thailand, and bat hunting is prohibited by law. A study showed NiV outbreak’s seasonal preference of cooler weather (below 17 °C) [47], for example it has been shown that the virus thrives better in urine at lower temperatures than at higher temperatures [48]. Apart from several days in January 2021, temperature in Chonburi does not often drop below 20 °C, and often averages above 30 °C which may help explain the lack of spillover to humans to date. However, this increases the risk of NiV outbreak as a result of the global climate change.

The risk of a NiV outbreak in Thailand is increasingly possible, as evidenced by the high identity shared between the NiV genome from Thai bats and the Bangladeshi patient, suggesting it is likely able to directly transmit to humans. The finding of NiV-infected bats trapped at the pig farms further raises concern of possible NiV transmission to pigs. Although there was no evidence of NiV infection in pigs in the same study, measures to prevent bats from coming into contact with pigs and avoidance of feeding pigs with partially eaten fruits must be actively promoted.

Study of community perceptions and knowledge of NiV transmission from bats in Bangladesh, underscores the need for educational interventions for targeted groups in the community [49]. Public awareness and willingness to steer clear of risk behaviors are vital to sustainably protect people in high-risk areas from NiV outbreak, and an opportunity for policy makers to strengthen public health infrastructure. This may include highlighting the ecosystem services and conservation of bats, so as to improve people’s current knowledge and subsequent behaviors regarding the role of bats in ecology and the spreading of NiV. The One Health effort conducted in Thailand for 20 years is building preparedness on three fronts: laboratory, community and the government. Conducting long-term longitudinal studies, such as the one in Chonburi province, has enabled Thai scientists to provide evidence-based information to policy decision makers while strengthening laboratory capacity and disease surveillance systems. Communities are being engaged at the village level to raise awareness of both the risks and benefits in living with bats. In Wat Luang village, researchers from the DNP, Kasetsart University and TRC-EID-HSC utilize “Living Safely with Bats”, a USAID-developed teaching aid tailored to the non-scientist population and Health Promoting Hospitals at the community level (translated and distributed by TRC-EID-HSC) [50].

## Conclusions

Thailand initiated NiV outbreak preparedness using the One Health approach prior to any NiV human or domestic animal outbreaks. This broad approach, encouraging inter-sectoral cooperation between human-animal-wildlife health sectors and raising awareness from the village up to the national government in Thailand is a successful example and model of a transboundary “One Health” approach for the Southeast Asian and South Asian region to monitor for any NiV outbreak, develop preventative measures, and be better prepared for the next pandemic.

## Supplementary Information

**Additional file 1: Table S1.** Accession numbers of the NiV used in this study (phylogenetic tree analysis of 357 bp). **Table S2.** PCR and ELISA IgG antibody results of pig specimens (nasal swab for PCR, serum for ELISA) collected from Chonburi and Prachinburi provinces, Thailand from August 2011 to November 2012. **Table S3.** PCR and ELISA IgG antibody results of specimens collected from healthy human volunteers from Wat Luang, Chonburi province, Thailand from 2010 to 2018.

## Data Availability

Genome sequences reported in this study have been deposited in GenBank under the accession numbers MW535746 (whole genome sequence) and MW573860- MW573880. Summary of the datasets used and/or analysed during the current study are included in this published article and its supplementary information files. Additional information may be obtained from the corresponding author on reasonable request.

## Abbreviations

BD: Bangladesh
BLAST: Basic Local Alignment Search Tool
bp: Base pairs
Bt: Bat
BWA: Burrows-Wheeler Aligner
cds: Coding sequence
CSF: Cerebrospinal fluid
DLD: Department of Livestock Development
DNP: Department of National Park, Wildlife and Plant Conservation
ELISA: Enzyme-linked immunosorbent assay
F gene: Fusion protein
G gene: Glycoprotein
GPS: Global Positioning System
GTR: General time-reversible
HL: *Hipposideros larvatus*
HU: Human
IgG: Immunoglobulin G
IN: India
KH: Cambodia
km: Kilometres
L gene: RNA Polymerase
M gene: Matrix protein
MMIA: Multiplex microsphere immunoassay
MOPH: Ministry of Public Health
MY: Malaysia
N gene: Nucleocapsid
NCBI: National Center for Biotechnology Information
NGMAP: Non-reference genome metagenomic analysis pipeline
NGS: Next generation sequencing
NIAH: National Institute of Animal Health
NiV: Nipah virus
NiV-BD: Nipah virus - Bangladesh strain
NiV-MY: Nipah virus - Malaysia strain
nt: Nucleotides
OD: Optical Density
P gene: Phosphoprotein
PG: *Pteropus giganteus*
PH: *Pteropus hypomelanus*
PI: Pig
PL: *Pteropus lylei*
PM: *Pteropus medius*
PV: *Pteropus vampyrus*
RNA: Ribonucleic acid
RT-PCR: Reverse transcription polymerase chain reaction
sG_tet_: Soluble tetrameric henipavirus receptor binding proteins
TH: Thailand
TRC-EID-HSC: Thai Red Cross Emerging Infectious Diseases Health Science Centre
USAID: United States Agency for International Development
US-CDC: Centers for Disease Control and Prevention, U.S.A.
WGS: Whole genome sequence
WHO: World Health Organization

## References

[1] Epstein JH, Anthony SJ, Islam A, Kilpatrick AM, Khan SA, Balkey MD (2020). Nipah virus dynamics in bats and implications for spillover to humans. Proc Natl Acad Sci.

[2] Wacharapluesadee S, Lumlertdacha B, Boongird K, Wanghongsa S, Chanhome L, Rollin P (2005). Bat Nipah virus, Thailand. Emerg Infect Dis.

[3] Luby SP (2013). The pandemic potential of Nipah virus. Antivir Res.

[4] Arunkumar G, Chandni R, Mourya DT, Singh SK, Sadanandan R, Sudan P (2019). Outbreak investigation of Nipah virus disease in Kerala, India, 2018. J Infect Dis.

[5] Chadha MS, Comer JA, Lowe L, Rota PA, Rollin PE, Bellini WJ (2006). Nipah virus-associated encephalitis outbreak, Siliguri, India. Emerg Infect Dis.

[6] Arankalle VA, Bandyopadhyay BT, Ramdasi AY, Jadi R, Patil DR, Rahman M (2011). Genomic characterization of Nipah virus, West Bengal, India. Emerg Infect Dis.

[7] World Health Organization (2004). Nipah virus outbreak (s) in Bangladesh, January–April 2004. Wkly Epidemiol Record= Relevé épidémiologique hebdomadaire.

[8] ICDDRB (2004). Person-to-person transmission of Nipah virus during outbreak in Faridpur District, 2004. Health Sci Bull.

[9] Olival KJ, Latinne A, Islam A, Epstein JH, Hersch R, Engstrand RC (2020). Population genetics of fruit bat reservoir informs the dynamics, distribution and diversity of Nipah virus. Mol Ecol.

[10] Wacharapluesadee S, Jittmittraphap A, Yingsakmongkon S, Hemachudha T. Chapter 53 Nipah Virus. In: Liu D, editor. Molecular detection of animal viral pathogens. 4th vol. Taylor & Francis Crc Press. pp. 455-66.

[11] Cappelle J, Hoem T, Hul V, Furey N, Nguon K, Prigent S (2020). Nipah virus circulation at human–bat interfaces, Cambodia. Bull World Health Organ.

[12] Breed AC, Meers J, Sendow I, Bossart KN, Barr JA, Smith I (2013). The distribution of henipaviruses in Southeast Asia and Australasia: is Wallace’s line a barrier to Nipah virus?. PLoS One.

[13] Reynes JM, Counor D, Ong S, Faure C, Seng V, Molia S (2005). Nipah virus in Lyle's flying foxes, Cambodia. Emerg Infect Dis.

[14] Plowright RK, Becker DJ, Crowley DE, Washburne AD, Huang T, Nameer PO (2019). Prioritizing surveillance of Nipah virus in India. PLoS Negl Trop Dis.

[15] Hasebe F, Thuy NT, Inoue S, Yu F, Kaku Y, Watanabe S (2012). Serologic evidence of nipah virus infection in bats, Vietnam. Emerg Infect Dis.

[16] Wacharapluesadee S, Samseeneam P, Phermpool M, Kaewpom T, Rodpan A, Maneeorn P (2016). Molecular characterization of Nipah virus from Pteropus hypomelanus in southern Thailand. Virol J.

[17] Clayton BA, Middleton D, Bergfeld J, Haining J, Arkinstall R, Wang L (2012). Transmission routes for Nipah virus from Malaysia and Bangladesh. Emerg Infect Dis.

[18] Baseler L, de Wit E, Scott DP, Munster VJ, Feldmann H (2015). Syrian hamsters (Mesocricetus auratus) oronasally inoculated with a Nipah virus isolate from Bangladesh or Malaysia develop similar respiratory tract lesions. Vet Pathol.

[19] Chua KB, Koh CL, Hooi PS, Wee KF, Khong JH, Chua BH (2002). Isolation of Nipah virus from Malaysian island flying-foxes. Microbes Infect.

[20] Halpin K, Hyatt AD, Fogarty R, Middleton D, Bingham J, Epstein JH (2011). Pteropid bats are confirmed as the reservoir hosts of henipaviruses: a comprehensive experimental study of virus transmission. Am J Trop Med Hyg.

[21] Rahman SA, Hassan L, Epstein JH, Mamat ZC, Yatim AM, Hassan SS (2013). Risk factors for Nipah virus infection among pteropid bats. Peninsular Malaysia Emerg Infect Dis.

[22] Escaffre O, Borisevich V, Rockx B (2013). Pathogenesis of Hendra and Nipah virus infection in humans. J Infect Dev Countries.

[23] Kasloff SB, Leung A, Pickering BS, Smith G, Moffat E, Collignon B (2019). Pathogenicity of Nipah henipavirus Bangladesh in a swine host. Sci Rep.

[24] Wacharapluesadee S, Boongird K, Wanghongsa S, Ratanasetyuth N, Supavonwong P, Saengsen D (2010). A longitudinal study of the prevalence of Nipah virus in Pteropus lylei bats in Thailand: evidence for seasonal preference in disease transmission. Vector-Borne Zoon Dis.

[25] Sukgosa N, Duangjai S, Duengkae P, Wacharapluesadee S, Songmongkol P, Yingsakmongkon S (2018). Genetic diversity and relationships among Lyle's flying fox colonies in Thailand. Agric Nat Resour.

[26] Weber N, Duengkae P, Fahr J, Dechmann DK, Phengsakul P, Khumbucha W (2015). High-resolution GPS tracking of Lyle's flying fox between temples and orchards in Central Thailand. J Wildl Manag.

[27] Thanapongtharm W, Linard C, Wiriyarat W, Chinsorn P, Kanchanasaka B, Xiao X (2015). Spatial characterization of colonies of the flying fox bat, a carrier of Nipah virus in Thailand. BMC Vet Res.

[28] Chaiyes A, Duengkae P, Wacharapluesadee S, Pongpattananurak N, Olival KJ, Hemachudha T (2017). Assessing the distribution, roosting site characteristics, and population of Pteropus lylei in Thailand. Raffles Bull Zool.

[29] Chaiyes A, Escobar LE, Willcox EV, Duengkae P, Suksavate W, Watcharaanantapong P (2020). An assessment of the niche centroid hypothesis: Pteropus lylei (Chiroptera). Ecosphere..

[30] Duengkae P, Srikhunmuang P, Chaiyes A, Suksavate W, Pongpattananurak N, Wacharapluesadee S (2019). Patch metrics of roosting site selection by Lyle’s flying fox (*Pteropus lylei* Andersen, 1908) in a human-dominated landscape in Thailand. Folia Oecolog.

[31] Thanapongtharm W, Paul MC, Wiratsudakul A, Wongphruksasoong V, Kalpravidh W, Wongsathapornchai K (2019). A spatial assessment of Nipah virus transmission in Thailand pig farms using multi-criteria decision analysis. BMC Vet Res.

[32] Wongnak P, Thanapongtharm W, Kusakunniran W, Karnjanapreechakorn S, Sutassananon K, Kalpravidh W (2020). A ‘what-if’scenario: Nipah virus attacks pig trade chains in Thailand. BMC Vet Res.

[33] Wacharapluesadee S, Hemachudha T (2007). Duplex nested RT-PCR for detection of Nipah virus RNA from urine specimens of bats. J Virol Methods.

[34] Li H, Durbin R (2009). Fast and accurate short read alignment with burrows–wheeler transform. Bioinformatics.

[35] Li H, Handsaker B, Wysoker A, Fennell T, Ruan J, Homer N (2009). The sequence alignment/map format and SAMtools. Bioinformatics..

[36] Milne I, Bayer M, Cardle L, Shaw P, Stephen G, Wright F (2010). Tablet—next generation sequence assembly visualization. Bioinformatics..

[37] Langmead B, Salzberg SL (2012). Fast gapped-read alignment with bowtie 2. Nat Methods.

[38] Katoh K, Misawa K, Kuma KI, Miyata T (2002). MAFFT: a novel method for rapid multiple sequence alignment based on fast Fourier transform. Nucleic Acids Res.

[39] Kumar S, Stecher G, Li M, Knyaz C, Tamura K (2018). MEGA X: molecular evolutionary genetics analysis across computing platforms. Mol Biol Evol.

[40] Rambaut A. FigTree v1.4.2. Institute of Evolutionary Biology, University of Edinburgh, Edinburgh. 2012. http://tree.bio.ed.ac.uk/software/figtree/.

[41] Schulz JE, Seifert SN, Thompson JT, Avanzato V, Sterling SL, Yan L (2020). Serological evidence for Henipa-like and filo-like viruses in Trinidad bats. J Infect Dis.

[42] Wacharapluesadee S, Ngamprasertwong T, Kaewpom T, Kattong P, Rodpan A, Wanghongsa S (2013). Genetic characterization of Nipah virus from Thai fruit bats (Pteropus lylei). Asian Biomed.

[43] Department of Livestock Development. Population of Pigs in Thailand in 2020. 2020. ICT Department. (http://ict.dld.go.th/webnew/images/stories/report/regislives/2020/T5-1-Pig.pdf)

[44] Conlan JV, Vongxay K, Jarman RG, Gibbons RV, Lunt RA, Fenwick S (2012). Serologic study of pig-associated viral zoonoses in Laos. Am J Trop Med Hygiene.

[45] Sendow I, Field HE, Adjid A, Ratnawati A, Breed AC, Morrissy C (2010). Screening for Nipah virus infection in West Kalimantan province. Indonesia Zoonoses Publ Health.

[46] Yong MY, Lee SC, Ngui R, Lim YA, Phipps ME, Chang LY (2020). Seroprevalence of Nipah virus infection in peninsular Malaysia. J Infect Dis.

[47] McKee CD, Islam A, Luby SP, Salje H, Hudson PJ, Plowright RK (2021). The ecology of Nipah virus in Bangladesh: a nexus of land-use change and opportunistic feeding behavior in bats. Viruses..

[48] Fogarty R, Halpin K, Hyatt AD, Daszak P, Mungall BA (2008). Henipavirus susceptibility to environmental variables. Virus Res.

[49] Hassan MM, Kalam M, Alam M, Shano S, Faruq AA, Hossain M (2020). Understanding the community perceptions and knowledge of bats and transmission of nipah virus in Bangladesh. Animals..

[50] PREDICT One Health Consortium 2018 (2018). Living safely with bats.

