# Supplementary material for: Two decades of one health surveillance of Nipah virus in Thailand
Source: One Health Outlook. 2021 Jul 5;3:12. doi: 10.1186/s42522-021-00044-9 (PMC8255096; doi:10.1186/s42522-021-00044-9)
Supplement: Supplementary file 1 — Additional file 1: Table S1. Accession numbers of the NiV used in this study (phylogenetic tree analysis of 357 bp). Table S2. PCR and ELISA IgG antibody results of pig specimens (nasal swab for PCR, serum for ELISA) collected from Chonburi and Prachinburi provinces, Thailand from August 2011 to November 2012. Table S3. PCR and ELISA IgG antibody results of specimens collected from healthy human volunteers from Wat Luang, Chonburi province, Thailand from 2010 to 2018. [file 42522_2021_44_MOESM1_ESM.docx]

**Supplementary Table 1.** Accession numbers of the NiV used in this study (phylogenetic tree analysis of 357 bp)

| **No.** | **Sequence Name** | **Sequence Name** | **Reference^†^** |
| --- | --- | --- | --- |
| 1 | KT163252.1 | KT163252.1 BtNiV/TH/PL/2011/3054 | [16] |
| 2 | DQ061853.2 | DQ061853.2 BtNiV/TH/PL/2003/UP61 | [2] |
| 3 | EU603734.1 | EU603734.1 BtNiV/TH/PL/2006/U1056 | [24] |
| 4 | KT163256.1 | KT163256.1 BtNiV/TH/PL/2011/33654 | [16] |
| 5 | KT163254.1 | KT163254.1 BtNiV/TH/PL/2010/15553 | [16] |
| 6 | MW573860 | MW573860 BtNiV/TH/PL/2009/2982 | This study |
| 7 | MW573861 | MW573861 BtNiV/TH/PL/2009/3276 | This study |
| 8 | MW573862 | MW573862 BtNiV/TH/PL/2012/55 | This study |
| 9 | MW573863 | MW573863 BtNiV/TH/PL/2012/161 | This study |
| 10 | MW573864 | MW573864 BtNiV/TH/PL/2012/184 | This study |
| 11 | MW573865 | MW573865 BtNiV/TH/PL/2016/B16P31 | This study |
| 12 | MW573866 | MW573866 BtNiV/TH/PL/2016/B16P32 | This study |
| 13 | MW573867 | MW573867 BtNiV/TH/PL/2017/B17602 | This study |
| 14 | MW573868 | MW573868 BtNiV/TH/PL/2017/B17606 | This study |
| 15 | MW573869 | MW573869 BtNiV/TH/PL/2017/B17640 | This study |
| 16 | MW573870 | MW573870 BtNiV/TH/PL/2017/B17641 | This study |
| 17 | MW573871 | MW573871 BtNiV/TH/PL/2018/B18018 | This study |
| 18 | DQ061854.2 | DQ061854.2 BtNiV/TH/PL/2003/UP101 | [2] |
| 19 | FJ648076.1 | FJ648076.1 BtNiV/TH/PL/2004/1248 | [39] |
| 20 | FJ648075.1 | FJ648075.1 BtNiV/TH/PL/2004/1184 | [39] |
| 21 | EF070190.1 | EF070190.1 BtNiV/TH/PL/2005/U53 | [29] |
| 22 | EF070183.1 | EF070183.1 BtNiV/TH/PL/2005/U8 | [29] |
| 23 | EU603754.1 | EU603754.1 BtNiV/TH/PL/2007/U2597 | [16] |
| 24 | EU603736.1 | EU603736.1 BtNiV/TH/PL/2006/U1190 | [16] |
| 25 | FJ648077.1 | FJ648077.1 BtNiV/TH/PL/2008/U2876 | [39] |
| 26 | KT163251.1 | KT163251.1 BtNiV/TH/PL/2010/1054 | [16] |
| 27 | MW573872 | MW573872 BtNiV/TH/PL/2018/B18019 | This study |
| 28 | EU603740.1 | EU603740.1 BtNiV/TH/PL/2006/U1242 | [24] |
| 29 | EU603751.1 | EU603751.1 BtNiV/TH/PL/2007/U2705 | [24] |
| 30 | FJ648080.1 | FJ648080.1 BtNiV/TH/PL/2008/U2905 | [39] |
| 31 | KT163255.1 | KT163255.1 BtNiV/TH/PL/2010/14653 | [16] |
| 32 | FJ648082.1 | FJ648082.1 BtNiV/TH/PL/2008/U2918 | [39] |
| 33 | KT163247.1 | KT163247.1 BtNiV/TH/PH/2010/1753 | [16] |
| 34 | KT163250.1 | KT163250.1 BtNiV/TH/PH/2011/8554 | [16] |
| 35 | EF070182.1 | EF070182.1 BtNiV/TH/PL/2005/U3 | [29] |
| 36 | KT163257.1 | KT163257.1 BtNiV/TH/PH/2011/18254 | [16] |
| 37 | DQ061851.2 | DQ061851.2 BtNiV/TH/HL/2002/SP20 | [2] |
| 38 | EU603726.1 | EU603726.1 BtNiV/TH/PL/2006/U561 | [16] |
| 39 | MW573873 | MW573873 BtNiV/TH/PL/2018/B18036 | This study |
| 40 | MW573874 | MW573874 BtNiV/TH/PL/2018/B18053 | This study |
| 41 | MW573875 | MW573875 BtNiV/TH/PL/2018/B18055 | This study |
| 42 | MW573876 | MW573876 BtNiV/TH/PL/2019/B19013 | This study |
| 43 | MW573877 | MW573877 BtNiV/TH/PL/2019/B19022 | This study |
| 44 | EF070189.1 | EF070189.1 BtNiV/TH/PL/2005/U48 | [29] |
| 45 | MW573878 | MW573878 BtNiV/TH/PL/2020/B20013 | This study |
| 46 | MW573879 | MW573879 BtNiV/TH/PL/2020/B20030 | This study |
| 47 | MW573880 | MW573880 BtNiV/TH/PL/2020/B20044 | This study |

**Supplementary Table 2** PCR and ELISA IgG antibody results of pig specimens (nasal swab for PCR, serum for ELISA) collected from Chonburi and Prachinburi provinces, Thailand from August 2011 to November 2012

| Specimen Collection Date | | Location | PCR results (positive/tested) | IgG antibody ELISA results (positive/tested) |
| --- | --- | --- | --- | --- |
| Year | **Month** |  |  |  |
| 2011 | August | Chonburi | 0/32 | 0/31 |
| 2012 | January | Chonburi | 0/12 | 0/12 |
|  |  | Prachinburi | 0/44 | 0/44 |
|  | April | Chonburi | 0/29 | 0/29 |
|  | July | Chonburi | 0/24 | 0/23 |
|  | October | Chonburi | 0/22 | 0/22 |
|  | November | Chonburi | 0/70 | 0/85 |
| Total | | | **0/233** | **0/246** |

**Supplementary Table 3** PCR and ELISA IgG antibody results of specimens collected from healthy human volunteers from Wat Luang, Chonburi province, Thailand from 2010 to 2018

| Date of specimen collection | Results (positive/tested) | | |
| --- | --- | --- | --- |
|  | **NiV PCR**  **from saliva** | **NiV PCR**  **from urine** | **IgG Antibody [detection method]** |
| November - December 2010 | N/A | N/A | 0/418 [ELISA] |
| May 2017 | 0/115 | 0/115 | N/A |
| May 2018 | 0/128 | 0/128 | 0/128 [Luminex] |
